# Deconstructing the Dissimilatory Sulfate Reduction Pathway: Isotope Fractionation of a Mutant Unable of Growth on Sulfate

**DOI:** 10.3389/fmicb.2018.03110

**Published:** 2018-12-14

**Authors:** Emma Bertran, William D. Leavitt, Andre Pellerin, Grant M. Zane, Judy D. Wall, Itay Halevy, Boswell A. Wing, David T. Johnston

**Affiliations:** ^1^Department of Earth and Planetary Sciences, Harvard University, Cambridge, MD, United States; ^2^Department of Earth Sciences, Dartmouth College, Cambridge, MD, United States; ^3^Department of Biological Sciences, Dartmouth College, Hanover, NH, United States; ^4^Department of Bioscience, Center for Geomicrobiology, Aarhus University, Aarhus, Denmark; ^5^Department of Biochemistry, University of Missouri, Columbia, SC, United States; ^6^Department of Environmental Sciences and Energy Research, Weizmann Institute of Science, Rehovot, Israel; ^7^Department of Geological Sciences, University of Colorado Boulder, Boulder, CO, United States

**Keywords:** chemostat, deletion mutant, metabolic pathway, sulfite reduction, sulfur isotope fractionation

## Abstract

The sulfur isotope record provides key insight into the history of Earth's redox conditions. A detailed understanding of the metabolisms driving this cycle, and specifically microbial sulfate reduction (MSR), is crucial for accurate paleoenvironmental reconstructions. This includes a precise knowledge of the step-specific sulfur isotope effects during MSR. In this study, we aim at resolving the cellular-level fractionation factor during dissimilatory sulfite reduction to sulfide within MSR, and use this measured isotope effect as a calibration to enhance our understanding of the biochemistry of sulfite reduction. For this, we merge measured isotope effects associated with dissimilatory sulfite reduction with a quantitative model that explicitly links net fractionation, reaction reversibility, and intracellular metabolite levels. The highly targeted experimental aspect of this study was possible by virtue of the availability of a deletion mutant strain of the model sulfate reducer *Desulfovibrio vulgaris* (strain Hildenborough), in which the sulfite reduction step is isolated from the rest of the metabolic pathway owing to the absence of its QmoABC complex (ΔQmo). This deletion disrupts electron flux and prevents the reduction of adenosine phosphosulfate (APS) to sulfite. When grown in open-system steady-state conditions at 10% maximum growth rate in the presence of sulfite and lactate as electron donor, sulfur isotope fractionation factors averaged −15.9‰ (1 σ = 0.4), which appeared to be statistically indistinguishable from a pure enzyme study with dissimilatory sulfite reductase. We coupled these measurements with an understanding of step-specific equilibrium and kinetic isotope effects, and furthered our mechanistic understanding of the biochemistry of sulfite uptake and ensuing reduction. Our metabolically informed isotope model identifies flavodoxin as the most likely electron carrier performing the transfer of electrons to dissimilatory sulfite reductase. This is in line with previous work on metabolic strategies adopted by sulfate reducers under different energy regimes, and has implications for our understanding of the plasticity of this metabolic pathway at the center of our interpretation of modern and palaeo-environmental records.

## 1. Introduction

The sulfur (S) isotopic composition of marine sedimentary sulfates (${\text{SO}}_{4}^{2-}$) and sulfides (H_2_S) encodes a composite of chemical and biological information on Earth's past sedimentary environments (Canfield, 2004). This record has been used extensively to identify major secular changes in Earth's surface conditions, including the initial rise of atmospheric oxygen (Farquhar et al., 2000; Habicht et al., 2002; Bekker et al., 2004), the Precambrian origin of different microbial metabolisms (Canfield, 1998; Johnston et al., 2005), and the onset of bioturbation in the early Paleozoic Era (Canfield and Farquhar, 2009; Tarhan et al., 2015). However, our capacity to infer paleo-environmental conditions depends heavily on an accurate and quantitative understanding of the mechanisms underpinning the sulfur cycle, and more specifically, those generating observable, and preservable isotope effects (Leavitt et al., 2013).

Decades of research identified microbial sulfate reduction (MSR) as a dominant metabolic pathway in the sulfur cycle (Canfield, 1998; Sim et al., 2011a; Leavitt et al., 2013). Sulfate reducers couple the reduction of sulfate to the oxidation of organic matter or molecular hydrogen (Canfield, 2004). The ultimate product of this metabolism is sulfide, as shown below in the reaction network 1.

(1) $${\text{SO}}_{4,extracellular}^{2-}⇌{\text{SO}}_{4,intracellular}^{2-}⇌\text{APS}⇌{\text{SO}}_{3}^{2-}⇌{\text{H}}_{2}\text{S},$$

In doing so, this metabolism carries a strong isotopic selectivity: it leaves the residual reactant (sulfate) enriched in ^34^S/^32^S, and the product (sulfide) depleted in ^34^S/^32^S. This isotopic biosignature captures physiological and environmental information at the time of its generation. Understanding the underlying biochemistry of this metabolism allows for the establishment of quantitative links between mass fluxes and isotope fractionation in intracellular and extracellular environments. These calibrations enable translation of observed isotopic enrichments and depletions into valuable environmental information.

Cellular scale calibrations of microbial sulfate reduction have revealed a cascade of additional factors known to exert some degree of control on isotope effects generated by MSR (Harrison and Thode, 1958). Such studies often target the physiological state of a microbe (Sim et al., 2011a,b; Leavitt et al., 2013) or the respiratory reaction pathway (Rees, 1973; Brunner and Bernasconi, 2005; Johnston et al., 2007), and have identified measurable and characteristic relationships between MSR fractionation factors and physiological and extracellular conditions, including net rates of sulfate reduction (Chambers et al., 1975; Goldhaber and Kaplan, 1980; Leavitt et al., 2013) as well as extracellular sulfate concentrations (Habicht et al., 2002, 2005). As for enzyme level fractionation, recent *in vitro* work isotopically characterized the dissimilatory sulfite reductase enzyme (DsrAB) - a key protein in the reductive pathway. This is a step toward a more detailed scaffold for the net MSR isotope effect (Leavitt et al., 2014). Complementary to this experimental work, theoretical approaches combining microbial and biochemical kinetics are shedding new light on the controls on isotopic fractionation at the cellular level. One approach in particular leans on aspects of reaction specific thermodynamics, intracellular redox potential, as well as enzyme catalysis and degree of saturation (Wing and Halevy, 2014). Overall, these fields bring together different, yet complementary information about isotopic fractionation during MSR. They highlight the intrinsic complexity of these organisms, their metabolic pathways, resulting fractionation factors, and underline a pressing need for a whole cell experimental approach to address the interplay of these different features. The level of theoretical sophistication noted above sets in place a road map for a merger with an equally sophisticated experimental program.

The current study focuses on the main and terminal reductive step within MSR - sulfite reduction to sulfide (Santos et al., 2015). To do so, we employ molecular genetics to quantify and model the sulfur isotopic fractionation capacity of a deletion mutant strain of the model sulfate reducer *Desulfovibrio vulgaris* (strain Hildenborough) (DvH) in open system conditions at steady state. This mutant of *D. vulgaris* is missing its QmoABC complex (ΔQmo), a key protein required for the reduction of **a**denosine **p**hospho**s**ulfate (APS) to sulfite (Zane et al., 2010). The ΔQmo mutant is incapable of reducing sulfate and instead uses sulfite as a terminal electron acceptor, in essence isolating the sulfite reduction step(s) from the rest of the MSR metabolic pathway, as shown in the reaction network below:

(2) $$SO_{3,out}^{2-}⇌SO_{3,in}^{2-}⇌H_{2}S.$$

As such, the ΔQmo will not metabolize sulfite the same way a wild-type sulfate reducer would. If a wild-type sulfate reducing bacterium is presented with sulfite, it will import, but since this substrate occupies a central place in the overall MSR network (see Equation 1), and owing to the reversible nature of the metabolism, back reaction to APS concomitant with sulfite reduction to sulfide is a possibility. This is eliminated in the ΔQmo mutant, so our approach is the cleanest expression of sulfite reduction.

We present results from a series of chemostat experiments run at 10% of maximum growth rate, chosen to assess conditions close to the upper limit of this cellular-scale isotope effect. While the resulting cell-specific sulfite reduction rates is by far not the lower limit for reported growth rates of sulfate reducers (Leavitt et al., 2013), implying near-equilibrium isotope fractionations are not necessarily reached, it will still allow, to a first order, approaching the upper limit of isotope effects during sulfite reduction and explore this step of the MSR reaction network. These results are then placed in the context of bacterial physiology and enzyme kinetics. This will be achieved by applying an established quantitative model, informed by both isotope equilibrium theory and microbial kinetics. We specifically explore the intrinsic isotope effects associated with sulfite reduction, reaction reversibility, and the degree to which isotopic equilibrium influences net cellular fractionation. This study establishes quantitative links between whole cell biochemistry, enzyme kinetics, and sulfur isotope effects that will further inform the interpretation of the geological sulfur isotope records.

## 2. Methods

### 2.1. Chemostat Experimental Set Up

The continuous culture experiment was performed in a chemostat (**chem**ical envir**o**nment in **stat**ic) at room temperature (21°C). The device included three inter-connected vessels. The central vessel (hereby referred to as the reactor) contained the bacteria growing in 0.5 L of continuously homogenized medium at constant pH (specifically, 7.2 ± 0.1), regulated by a pH probe-activated titration pump (see Supplementary Material for details). For these experiments, lactate was the limiting substrate (10 mM) while sulfite was in excess (20 mM), both were delivered at a controlled rate. This choice was informed by the 2:1 (lactate:sulfate) stoichiometric ratio of coupled lactate oxidation and sulfate reduction (Keller and Wall, 2011): offsetting that ratio to 1:2 (lactate:sulfite) ensured lactate limitation. Both gas and liquid outflow were captured in separate 20% (w/v) zinc acetate solution and 1% (w/v) zinc chloride (ZnCl_2_) solution (hereafter referred to as the gas and liquid trap, respectively). Rates of inflowing and outflowing medium were regulated by a single pump to maintain a constant volume in the reactor. All components of the reactor were autoclave sterilized before the start of the experiment. Prior to initiating steady-state growth, the ΔQmo mutant was allowed to grow in batch conditions and reach previously determined mid-exponential OD_600_ levels.

### 2.2. Maximum Specific Growth Rate Determinations

The maximum specific growth rate (μ_*max*_) of the strain was first determined in batch culture in the same growth medium as used for the chemostat, and relates to cellular doubling time:

(3) $$doubling=\frac{ln(\frac{N_{f}}{N_{i}})}{ln(2)}$$

where N_*f*_ and N_*i*_ are cell densities at the beginning and end of exponential phase (Crozat et al., 1991). This gives the doubling rate per day. From this batch culture work, a μ_*max*_ of 3.6 day^−1^ was obtained for the ΔQmo mutant. Chemostat experiments were conducted at 10% of μ_*max*_, or 0.36 day^−1^. The dilution rate (D) (Crozat et al., 1991) is determined as following then:

(4) $$D=\frac{\frac{L}{day}}{L}=\frac{1}{day}$$

Therefore, for a reaction volume of 0.5 L, the resulting dilution rate D for the experiment is 0.18 $\frac{L}{d}$.

### 2.3. Sampling Scheme

Over the course of the experiment, the reactor, liquid and gas traps were sampled at regular time intervals for chemical and biological quantification. These measurements include cell densities (as OD_600_, measured in real time and later converted to cell counts), sulfur chemistry (concentration and isotopic composition of sulfite, sulfide, and thiosulfate: samples from the reactor were preserved with an anoxic solution of 0.1% ZnCl_2_ prior to storage) and carbon species (lactate and acetate: samples from the reactor and the liquid trap were combined with 600 mM formaldehyde solution prior to storage). All samples were preserved at −20°C prior to sample treatment for analysis. The details of chemical and isotopic analyses are presented in the Supplemental Material and are outlined elsewhere (Leavitt et al., 2013, 2014).

### 2.4. Sulfur Isotopic Composition: Analytical Procedure

All sulfur species - both reduced and oxidized moities - were converted to silver sulfide (Ag_2_S) (see Johnston et al., 2007; Leavitt et al., 2014). Following this, samples were fluorinated under 10X excess of F_2_ headspace at 250°C, generating analyte sulfur hexafluoride (SF_6_). SF_6_ was then purified cryogenically via distillation at −117°C and chromatographically on a 6-foot molecular sieve 5 Å column coupled to a 6-foot HayeSep Q 1/8-inch column, with a TCD for detection and quantification. Purified SF_6_ was measured as ${\text{SF}}_{5}^{+}$ (m/z of 127, 128, 129, and 131) on a Thermo Scientific MAT 253 (1σ: δ^34^S ± 0.2 ‰, △^33^S ± 0.006 ‰, △^36^S ± 0.2 ‰).

### 2.5. Isotope Ratio and Fractionation Calculations

The isotopic composition of a given sulfur phase is expressed using the ‰ difference in the phase's isotopic ratio and the Canon Diablo Troilite Standard. For major sulfur isotopes:

(5) $$\delta^{3x}S=(\frac{{\;}^{3x}R_{sample}}{{\;}^{3x}R_{standard}}-1)\times 1000,$$

with x = 3, 4, or 6, and where isotope ratios (^3*x*^R) are:

(6) $${\;}^{3x}R=\frac{{\;}^{3x}r}{{\;}^{32}r}\;.$$

The isotopic composition of each sulfur species can be normalized to the composition of incoming media sulfite, thereby expressing net fractionation factors ε in units of ‰:

(7) $${\;}^{34}\varepsilon_{x/sulfite}=1000*\left(\frac{{\;}^{34}R_{x}}{{\;}^{34}R_{sulfite-media}}-1\right).$$

In relating two pools (A and B), an alpha value (^3*x*^α), or fractionation factor, is defined:

(8) $${\;}^{3x}\alpha_{A-B}=\;\frac{{\;}^{3x}R_{A}}{{\;}^{3x}R_{B}}=\frac{(\delta^{3x}S_{A}/1000)+1}{(\delta^{3x}S_{B}/1000)+1}$$

In complement to conventional major isotope fractionation effects, minor sulfur isotope fractionation is also quantifiable via:

(9) $${\;}^{33}\lambda =\frac{ln\left(\frac{{\;}^{33}R_{sulfite}}{{\;}^{33}R_{sulfite}}\right)}{ln\left(\frac{{\;}^{34}R_{sulfite}}{{\;}^{34}R_{sulfite}}\right)}$$

## 3. Results

The chemostat reached steady state (chemically and biologically) after a period of 400 h and was sampled five times (V = 10 mL) over the ensuing 100 h (identified as a-e, for reference). The biomass stabilized at a cell density of 2.16 × 10^8^ cells per mL (1 σ = 1.6 × 10^6^ cells per mL). The pH held constant at 7.2. Sulfite and thiosulfate in the reactor averaged to 16.5 mM (1 σ = 0.8 mM) and 0.5 mM (1 σ = 0.1 mM), respectively. Sulfide, derived from the gas trap, averaged to 10 mM (1 σ = 3 mM). Acetate levels held steady at 7.9 mM (1 σ = 0.9 mM), with lactate remaining below detection. This last point is consistent with lactate serving as the limiting substrate (see Figure S1 in the Supplementary Material).

The constancy in the concentrations and isotopic compositions, as well as the absence of lactate in the reactor, confirm steady state conditions were reached and maintained over the reported time interval. The balance of the flux of sulfur entering and leaving the reactor for each sampling point is shown in Figure S2 in the Supplementary Material.

**Figure 1:**
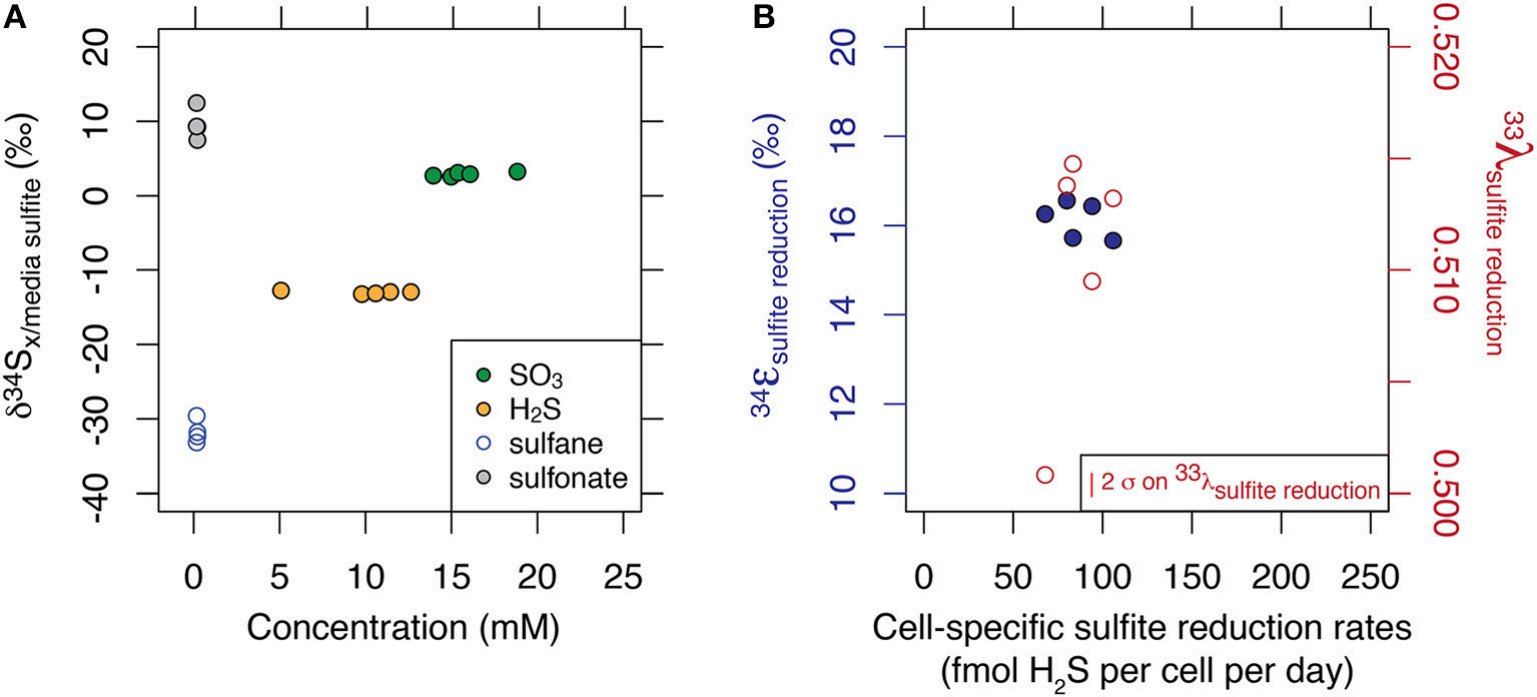
Multiple sulfur isotope data of each sulfur substrate generated at steady state and at 10% of maximum growth. Major isotopic compositions are shown relative to the composition of incoming sulfite, and as a function of their respective concentrations **(A)**. Fractionation factors associated with the reduction of sulfite to sulfide are shown for major and minor isotope systematics **(B)**. Calculation of the error in λ scales with ε and is described in Johnston et al. (2007).

While the ${\;}^{34}\varepsilon_{sulfide/sulfite}$ values averaged to −15.2 ‰ (1 σ = 0.2), thiosulfate presented a large site-specific isotopic offset of up to 40 ‰ between the sulfane and sulfonate sites. The site-specific thiosulfate isotope data is consistent with sulfane and sulfonate production based on an original sulfite valence state. Results are displayed in Figure 1A. Minor isotope effects (^33^λ) ranged from 0.5008 to 0.5147, and results are displayed in Figure 1B.

## 4. Discussion

Isotopic fractionation is useful in diagnosing environmental change, and sulfur isotopes, as they relate to MSR, are key in these reconstructions. In order to better understand the controls and generation of these isotope effects, studies have taken numerous approaches, examining rate relationships (Leavitt et al., 2013), changing the nature of the sulfur-bearing substrate (e.g., sulfite and thiosulfate, as opposed to sulfate, Leavitt et al., 2014), and even probing the fractionation consequences of *in vitro* enzymes (DsrAB in Leavitt et al., 2015). Here, we take those approaches down a different path via the genetic mutant the ΔQmo mutant, which is incapable of growth on sulfate. The removal of the QmoABC complex disrupts electron flux and leaves this *D. vulgaris* mutant as a strict sulfite reducer, isolating this subset of MSR biochemistry.

### 4.1. The ΔQmo Mutant Isotopic Signature

**Figure 2:**
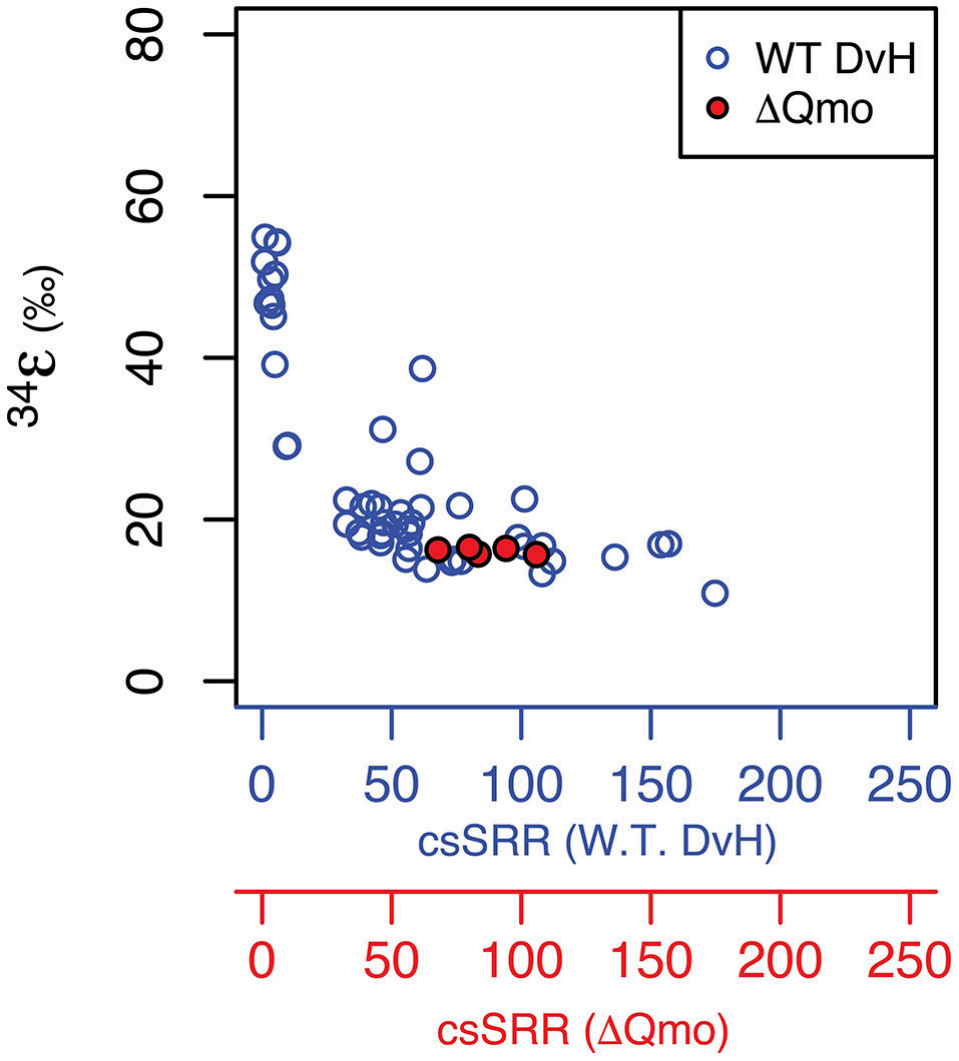
Major isotope fractionation factor between *reactant* and *product* as a function of rate of sulfur substrate reduction produced by both the wild-type (specifically, cell-specific *sulfate* reduction rates, blue circles and blue axis Leavitt et al., 2013) and mutant strains of *Desulfovibrio vulgarirs* (this study, hence specifically cell-specific *sulfite* reduction rates, red circles and red axis). Units are the same as for Figure 1.

Growth as a sulfite reducer, as a function of rate, is related to wild type growth. As captured in Figure 2, the produced major sulfur isotope effects overlap between sulfate and sulfite reduction at equivalent growth rates. This suggests sulfite reduction might significantly contribute to net sulfur isotope fractionation during sulfate reduction at higher growth rates. Further, the observed major sulfur isotope effect for the ΔQmo mutant is within the range of sulfite reduction by a number of sulfate reducing bacteria strains (Leavitt et al., 2015 and references within). However, direct comparison should be taken with a grain of salt, due to the different reaction networks for sulfite reduction found in the ΔQmo mutant used here, and a fully constituted wild-type strain of *D. vulgaris*. The latter will indeed not perform sulfite reduction in a manner equivalent to the ΔQmo mutant since, in the wild-type strain, there is the possibility of sulfite oxidation to APS. This backflux is isotopically important (Johnston et al., 2007; Leavitt et al., 2013; Wing and Halevy, 2014) but is not accessible, or expressed in the ΔQmo mutant. Our observed net fractionation during sulfite reduction is in contrast to strict predictions from previous work (Wing and Halevy, 2014), however it could also be a remnant of similar fractionations between APS reduction (the control in Wing and Halevy, 2014) and DsrAB/C. This can be further interrogated via a quantitative treatment of the ΔQmo mutant data.

We thus set to explore the isotopic consequences of life as a strict sulfite reducer. As shown in Figure S2 in the Supplementary Material, sulfur mass balance is completely closed over the course of the chemostat experiment. This indicates a lack of sulfate production during growth of the ΔQmo mutant in the presence of sulfite and lactate, and thus of intracellular sulfite oxidation to APS, and all the way back to sulfate. This supports the fact that we are, indeed, analyzing the growth and isotopic signature of a strict sulfite reducer in open system conditions. As noted above and shown in Table 1 in Supplementary Material, the mean ^34^ε for sulfite reduction is −15.2 ± 0.2 ‰, with a minor isotopic fractionation of 0.510 ± 0.006 and 1.89 ± 0.03 for ^33^λ and ^36^λ, respectively. In the case of ^33^λ, there is an outlier that is analytically defendable but quite low in value relative to previous measurements. Without this measurement, the mean ^33^λ value increases and error tightens (0.512 ± 0.002). This observed isotopic signature, produced in a highly constrained environment, will serve as calibration for understanding the biochemistry of sulfite reduction.

### 4.2. The Sulfite Reduction Metabolism

The chemostat experiment presented above captured net sulfite reduction to sulfide. While trace amounts of thiosulfate were observed, it did not represent a quantitatively significant sulfur pool, and thus will not be included in the following discussion. That noted, and consistent with earlier works (Leavitt et al., 2014), there are large and potentially significant site-specific effects that would require consideration in experiments or environments where thiosulfate plays a quantitatively significant role. Moving forward, the net reaction can hence be simplified to include sulfite uptake by the cell, and its subsequent reduction to sulfide. The latter comes at the expense of electrons, delivered by an electron carrier.

From here on, our general reaction then becomes:

(10) $$SO_{3,out}^{2-}+2H^{+}⇌SO_{3,in}^{2-}+EC_{red}+2H^{+}⇌H_{2}S+EC_{ox}$$

Where EC_*red*/*ox*_ refers to an electron carrier, either in the reduced or oxidized state.

Experimental and theoretical evidence for the biochemistry of sulfite uptake is scarce. However, sulfite and sulfate have similar structures and net charges. It would be logical to infer that sulfite uptake should take place via the same symporters as those used for the uptake of sulfate, resulting in a similar proton gradient for both sulfate and sulfite uptake.

The identity of the electron carrier involved during the reduction of sulfite is crucial. This reductive step is catalyzed by the enzyme dissimilatory sulfite reductase (DsrAB/C), which is fully constituted in the ΔQmo mutant. Recall that it is a deletion mutant of *D. vulgaris* strain Hildenborough, differing from the wild type strain only in its incapacity to use sulfate as electron acceptor. However, the interaction of DsrAB/C with a suite of possible electron carriers is still uncertain. During sulfite reduction to sulfide, a total of six electrons are required, transferred in two steps by a specific electron carrier. The first transfer is to the catalytic site of DsrAB, and reduces sulfite to an enzyme-bound S(II) intermediate (Santos et al., 2015), and the electron carrier promoting this reaction has yet to be identified. The second transfer reduces this intermediate (after DsrC binds to the catalytic site of DsrAB and forms a DsrC-bound zero valence S trisulfide product) to sulfide and DsrC (Santos et al., 2015). This reaction is known to be catalyzed, at least in part, by membrane-bound electron carrier complex DrsMKJOP. To do so, the latter complex oxidizes menaquinol. However, other electron carriers have been proposed to perform the same step (Venceslau et al., 2010; Santos et al., 2015). A number of electron carriers have been identified in sulfate reducing bacteria, including the common ferredoxin and menaquinone complexes (Tindall et al., 1989). In addition, menaquinones are membrane-bound, and can be directly involved in energy conservation. For these reasons, these electron carriers have been the focus of recent biochemical work attempting to establish a direct physiological role of these complexes during sulfate reduction (Ramos et al., 2012; Price et al., 2014). However, other electron carriers have also been identified in sulfate reducing organisms, including rubredoxin, flavodoxin, cytochrome c3 and rubrerythrin (Odom and Peck, 1984; Kremer et al., 1988; Ramos et al., 2012; Price et al., 2014; Rabus et al., 2015; Dorries et al., 2016), as well as transmembrane redox complexes, such as high-molecular-weight cytochrome (Hmc), tetraheme membrane cytochrome complex (Tmc), octaheme cytochrome complex (Ohc) or ferredoxin/flavodoxin-NAD^+^ reductase (Rnf), and flavoredoxin.

#### 4.2.1. Isotopic Consequences of Sulfite Reduction

We aim at refining our understanding of the cellular biochemistry of sulfite uptake and its subsequent reduction. Our specific goals are to determine (1) the identity of the electron carrier transferring electrons to DsrAB/C, and (2) whether the biochemistry of sulfite uptake significantly differs from that of sulfate uptake. For this we will use the sulfur isotopic signature produced by the ΔQmo mutant strain −as it will be a balanced contribution between the isotope effects associated with sulfite uptake and its reduction-, together with a quantitative model that establishes direct links between net sulfur isotope effects, cell-specific rates of sulfate reduction, and thermodynamics to derive the parameters needed to answer each of these questions.

Traditionally, sulfur isotope signatures are used to assess the degree of reversibility of individual enzymatic reactions using a framework that is formalized in simple box models (Johnston et al., 2005, 2007). Recently, Wing and Halevy (Wing and Halevy, 2014) merged enzymatics with thermodynamics for a finer understanding of the operation of the full sulfate reduction network. We use a simplified version of this model to reflect the biochemistry captured in equation 10, tailored for isotopic investigation. Keeping with the published formalism (Wing and Halevy, 2014), sulfite reduction is modeled as:

(11) $$SO_{3,out}^{2-}\overset{{\;}^{3x}\alpha_{1},J_{1}}{\underset{{\;}^{3x}\alpha_{2},J_{2}}{⇌}}SO_{3,in}^{2-}\overset{{\;}^{3x}\alpha_{3},J_{3}}{\underset{{\;}^{3x}\alpha_{4},J_{4}}{⇌}}H_{2}S.$$

In the following, subscripts *p* and *r* denote product and reactant, respectively. Net fractionations ($\alpha_{r,p}^{net}$) are captured by a balance of equilibrium and kinetic isotope effects ($\alpha_{r,p}^{eq}$ and $\alpha_{r,p}^{kin}$), modulated by relative mass fluxes. This mass flux (or the J_*x*_ terms in Equation 11) is simplified as the relative backward to forward flux, expressed thereafter as *f*. This term reflects the degree of reversibility of the reaction itself. When *f* approaches zero, the reaction is considered unidirectional (in the forward direction) and irreversible. Conversely, when *f* approaches unity, forward and backward fluxes are equal, the reaction is completely reversible, and by definition at chemical and isotopic equilibrium.

Under steady state conditions, the net fractionation for the reaction of sulfite to sulfide, the last step in our reaction network, is therefore captured as:

(12) $$\begin{array}{l} {\;}^{34}\alpha_{SO3,in-H2S}^{net}{=}^{34}\alpha_{SO3,in-H2S}^{kin}+f_{SO3,in-H2S}\\ \;\times {[}^{34}\alpha_{SO3,in-H2S}^{eq}{-}^{34}\alpha_{SO3,in-H2S}^{kin}]. \end{array}$$

Values for ${\;}^{3x}\alpha_{SO3,in-H2S}^{eq}$ have been calculated based on thermodynamic principles (Johnston et al., 2007), yielding values of 0.952, 0.515, and 1.89 for the ${\;}^{34}\alpha_{SO3,in-H2S}^{eq}$, ${\;}^{33}\lambda_{SO3,in-H2S}^{eq}$, and ${\;}^{36}\lambda_{SO3,in-H2S}^{eq}$, respectively at 25°C - the temperature of our chemostat reaction. Kinetic fractionation effects are dominated by the dissimilatory sulfite reductase enzyme (DsrAB/C), and are derived from experimental approaches (Leavitt et al., 2015). The intrinsic DsrAB/C fractionation was recently calibrated via enzyme extract experiments, yielding values of 0.984, 0.515 and 1.89 for the ${\;}^{34}\alpha_{SO3,in-H2S}^{kin}$, ${\;}^{33}\lambda_{SO3,in-H2S}^{kin}$, and ${\;}^{36}\lambda_{SO3,in-H2S}^{kin}$ at 25°C, respectively (Wing and Halevy, 2014). The final term, *f*_*SO*3, *in*−*H*2*S*_, is the ratio of backward to forward reaction between intracellular sulfite and product sulfide.

Following the same approach as for sulfite reduction, the expression for net isotope effects associated with the uptake of sulfite becomes:

(13) $$\begin{array}{l} {\;}^{34}\alpha_{SO3,out-SO3,in}^{net}{=}^{34}\alpha_{SO3,out-SO3,in}^{kin}+f_{SO3,out-SO3,in}\\ \;\times {[}^{34}\alpha_{SO3,out-SO3,in}^{eq}{-}^{34}\alpha_{SO3,out-SO3,in}^{kin}], \end{array}$$

where fractionation factors now reflect the isotopic equilibrium and kinetics associated with sulfite uptake from the extracellular environment into the cell. As the nature of the sulfoxy anion has not changed, the ${\;}^{34}\alpha_{SO3,out-SO3,in}^{eq}$ is set to equal 1. On the other hand, ${\;}^{34}\alpha_{SO3,out-SO3,in}^{kin}$ carries a value of 1.003, as measured in pure cell extracts of *Desulfovibrio desulfuricans* during *sulfate uptake* (Harrison and Thode, 1958). We defend this choice based on structural and charge similarities between sulfite and sulfate, and a lack of explicit sulfite data for the kinetic isotope effect associated with its uptake by sulfate reducing bacteria. The *f*_*SO*3, *out*−*SO*3, *in*_ term then defines exchange rates across the membrane.

We nest Equations (12) and (13) and yield a full expression for the isotopic consequences of metabolic sulfite reduction:

(14) $$\begin{array}{l} 34\alpha_{SO3,out-H2S}^{net}{=}^{34}\alpha_{SO3,out-SO3,in}^{kin}+f_{SO3,out-SO3,in}\\ \times {[}^{34}\alpha_{SO3,out-SO3,in}^{eq}\times^{34}\alpha_{SO3,in-H2S}^{net}{-}^{34}\alpha_{SO3,out-SO3,in}^{kin}]. \end{array}$$

First order analysis of Equation (14) identifies four useful end-members, set by the *f* terms, that will guide the discussion. Recall that when *f* equals 1, the isotopic solution approaches equilibrium. Similarly, when *f* equals zero, the isotopic solution approaches the kinetic fractionation.

When both *f*_*SO*3, *out*−*SO*3, *in*_ and *f*_*SO*3, *in*−*H*2*S*_ reach unity, each step in the reaction network is at, or very near equilibrium, and as a result the entire solution is near equilibrium. Since the uptake equilibrium was taken to be 1, net fractionation will be dominated by the *sulfite-sulfide equilibrium*. When the value of *f*_*SO*3, *in*−*H*2*S*_ approaches zero - while keeping *f*_*SO*3, *out*−*SO*3, *in*_ equal to 1 − the *sulfite reduction step* becomes the *rate controlling step*. The isotopic effect associated with sulfite reduction is purely kinetic, and net fractionation will approach but will not exceed the kinetic fractionation associated with DsrAB/C. If this distribution of overall metabolic rate control is reversed, that is, if *f*_*SO*3, *out*−*SO*3, *in*_ approaches zero and *f*_*SO*3, *in*−*H*2*S*_ is set equal to 1, the net fractionation will reflect the *kinetic fractionation* exhibited by *sulfite uptake alone*.

Intermediate net fractionations can also be produced when the control on rate is shared between the two steps in the reaction network -when both *f*_*SO*3, *out*−*SO*3, *in*_ and *f*_*SO*3, *in*−*H*2*S*_ take any value larger than 0 and lower than 1, with no single step exerting total control. This relationship is derived by reorganizing the equations above as:

(15) $$f_{SO3,out-SO3,in}=\;\frac{\alpha_{SO3,out-H2S}^{net}-\alpha_{SO3,out-SO3,in}^{kin}}{\alpha_{SO3,out-SO3,in}^{eq}[(\alpha_{SO3,in-H2S}^{eq}-\alpha_{SO3,in-H2S}^{kin})f_{SO3,in-H2S}+\alpha_{SO3,in-H2S}^{kin}]-\alpha_{SO3,out-SO3,in}^{kin}}.$$

**Figure 3:**
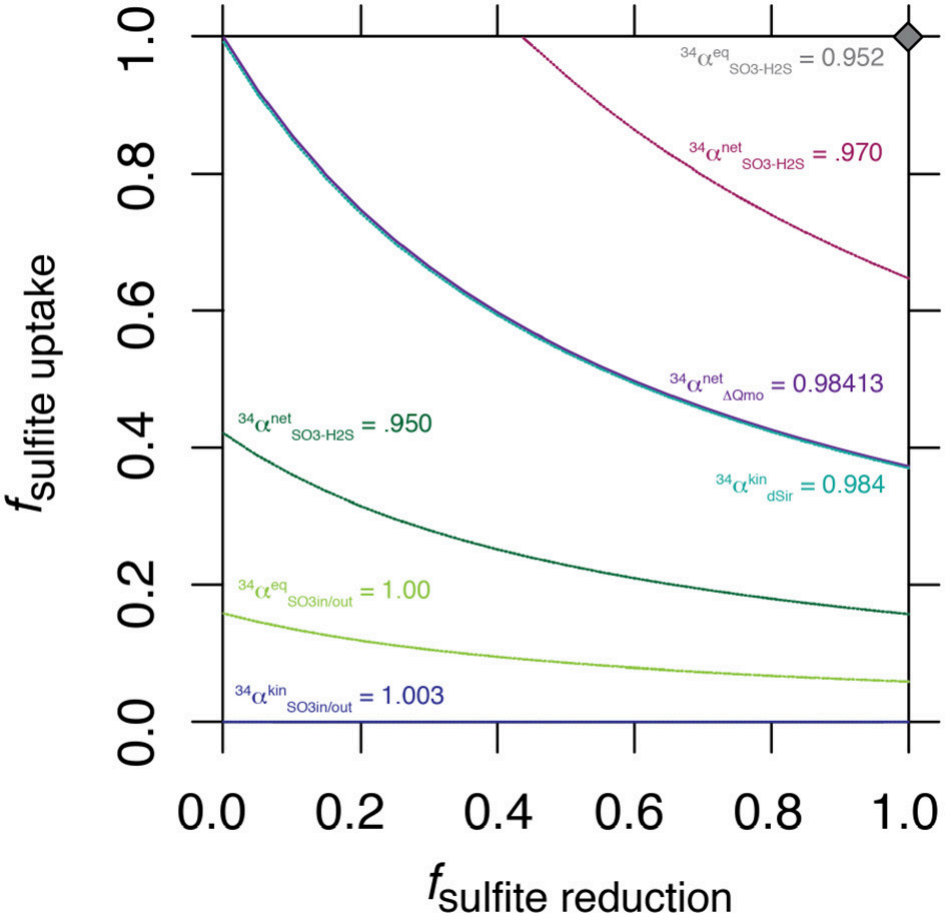
Solution space for the values of *f*_*uptake*_ and *f*_*SO*3, *in*−*H*2*S*_ for a range of synthetic ${\;}^{34}\alpha_{sulfite,out-sulfide}^{net}$, including the value generated by ΔQmo mutant experiments.

The nature of this *shared control* is evident through a synthetic example presented in Figure 3, where a range of synthetic ${\;}^{34}\alpha_{SO3,out-H2S}^{net}$ is shown for a range of values for *f*_*SO*3, *in*−*H*2*S*_.

The intriguing aspect of our whole-organism results is their similarity to the kinetic isotope effect recently measured for naked dissimilatory sulfite reductase (dSir) (Leavitt et al., 2015). This implies that *f*_*SO*3, *out*−*SO*3, *in*_ is near 1, and *f*_*SO*3, *in*−*H*2*S*_ is near 0. It follows that the observed net sulfur isotope effect is effectively equal to the isotope effect associated with dSir. In this case, the resemblance between the high rate fractionation values produced during MSR and those for the ΔQmo mutant (Figure 2) could either be coincidental, or require that the steps upstream from sulfite reduction in a fully constituted MSR reaction network are fully reversible as well. Here we explore situations that do not require these end-member assumptions and we explore how, as the net fractionation swings from an inverse isotope effect of 1.003 toward a normal isotope effect of 0.952, different combinations of f terms can accommodate the solution (Figure 3). Also included in Figure 3 is a line of constant ${\;}^{34}\alpha_{sulfite,out-sulfide}^{net}$ equal to the values generated within the chemostat studies presented above. The solution space that most uniquely represents our system (${\;}^{34}\alpha_{sulfite,out-sulfide}^{net}$ = 0.984) bears strong similarities to the case when net fractionations are set equal to the kinetic isotopic effect associated with sulfite reduction (noted as dsir on Figure 3). This reflects the similarity between the experimental results of the ΔQmo mutant experiment and that with pure DsrAB (Leavitt et al., 2015). With this relationship in hand, the value of *f*_*SO*3, *out*−*SO*3, *in*_ can be directly calculated for a given *f*_*SO*3, *in*−*H*2*S*_. In doing so, the inferred value for degree of reversibility during sulfite uptake is effectively constrained by the net isotope effect produced by the mutant strain, as long as reaction reversibility during sulfite reduction is well understood.

As depicted earlier, the electron donor during sulfite reduction has not been definitely identified, a difference that bears significant consequences for the reaction itself. Using the Wing and Halevy model (Wing and Halevy, 2014), we explore the effect of varying electron red/ox pair on the degree of reversibility of sulfite reduction. We then use the constrains on the resulting *f*_*SO*3, *out*−*SO*3, *in*_ described above to explore the biochemistry of sulfite uptake, and guide our interpretation on the most likely identity of the electron donor during sulfite reduction.

### 4.3. Refining the Biochemistry of Sulfite Reduction

Reaction reversibility is related to the energetics of the reaction itself at the standard state, the temperature of the system, and the concentration of metabolites involved:

(16) $$f_{p,r}=\frac{\Pi_{i}{\left[p_{i}\right]}^{m_{i}}}{\Pi_{j}{\left[r_{j}\right]}^{n_{j}}}e^{\Delta G^{{}^{\circ}}/RT},$$

where [r_*j*_] is the concentration of reactant *j, n*_*j*_ is the stoichiometry of said reactant. Similarly [p_*i*_] is the concentration of product *i*, and *m*_*i*_ is the stoichiometry of that product. ΔG° is the standard Gibbs free energy of the reaction, and R and T are the gas constant, and the temperature of the system, respectively.

#### 4.3.1. Sulfite Reduction

Per reaction 10 and Equation 16, *f*_*SO*3, *in*−*H*2*S*_ depends on the biochemical parameters specific to the choice of electron donor performing sulfite reduction. These include the standard Gibbs free energy of the reaction, and the relative abundance of reduced to oxidized electron carriers, as evident in Equation (17):

(17) $$f_{SO3,in-H2S}=\frac{[H_{2}S]{\left[EC_{ox}\right]}^{3}}{[SO_{3}^{2-}]{\left[EC_{red}\right]}^{3}}e^{\Delta G_{reduction}^{o}/RT},$$

where [EC_*ox*_] and [EC_*red*_] are the concentrations of oxidized and reduced electron carriers, respectively, and Δ${\text{G}}_{reduction}^{o}$ is the standard free Gibbs energy during the reduction of sulfite. These are electron carrier-specific, so we explore their effect on *f*_*SO*3, *in*−*H*2*S*_ (Figure S3 in the Supplementary Material). With these, in combination with equation 15, we directly calculate the values for *f*_*SO*3, *out*−*SO*3, *in*_ given net sulfur isotope effects produced by ΔQmo mutant across the same space of ΔG° and electron carrier red/ox ratios (Figure S5 in the Supplementary Material).

#### 4.3.2. Sulfite Uptake

Sulfite uptake has its own set of biochemical controls, however these are not well defined. While the above analysis allowed determining the values for *f*_*SO*3, *out*−*SO*3, *in*_, we did not enhance our understanding of the biochemistry of sulfite uptake. To do so, we apply the same approach as for *f*_*SO*3, *in*−*H*2*S*_ to *f*_*SO*3, *out*−*SO*3, *in*_, this time to explore the effect of maximal metabolic rate of the reaction, and half saturation constant. We use the following relationship:

(18) $$\begin{array}{l} J_{uptake}=V_{uptake}^{max}(\frac{{\left[SO_{3}^{2-}\right]}_{out}/K_{As}}{1+{\left[SO_{3}^{2-}\right]}_{out}/K_{As}+{\left[SO_{3}^{2-}\right]}_{in}/K_{Ap}})\\ \;\times (1-\frac{{\left[SO_{3}^{2-}\right]}_{in}{\left[H^{+}\right]}_{in}^{n}}{{\left[SO_{3}^{2-}\right]}_{out}{\left[H^{+}\right]}_{out}^{n}}e^{\Delta G_{uptake}^{o}/RT}), \end{array}$$

where J_*uptake*_ is the net uptake of sulfite into the intracellular environment, ${\text{V}}_{uptake}^{max}$ the maximal metabolic rate of the reaction, and K_*As*_ and K_*Ap*_ the half-saturation constants for the substrate (extracellular sulfite) and product (intracellular sulfite) of the reaction itself, which are set equal to each other. Δ${\text{G}}_{uptake}^{o}$ is the standard Gibbs free energy of the reaction, which is assumed to be the same as for sulfate uptake. Given the similar charge in sulfite and sulfate, it is fair to assume the same number of protons (*n* in the exponent of ${\text{H}}_{in}^{+}$/${\text{H}}_{out}^{+}$) will be transported during sulfite uptake than during the uptake of sulfate. These were constrained in the model for a fully constituted sulfate reducer (Wing and Halevy, 2014), where this number depends on extracellular pH, the proton motive force, membrane potential, and calibrated over a range of extracellular sulfate levels. The model assumes steady state conditions, thus J_*uptake*_ is equal to the net rate of sulfite reduction for the reaction network (Wing and Halevy, 2014). The expression for *f*_*SO*3, *out*−*SO*3, *in*_ is already included in Equation (18). Indeed, the degree of reversibility of the uptake step is defined as:

(19) $$f_{SO3,out-SO3,in}=\frac{{\left[SO_{3}^{2-}\right]}_{in}{\left[H^{+}\right]}_{in}^{n}}{{\left[SO_{3}^{2-}\right]}_{out}{\left[H^{+}\right]}_{out}^{n}}e^{\Delta G_{uptake}^{o}/RT}.$$

This allows exploring the effect of the maximal metabolic rate of the reaction and the half-saturation constant of the reaction on *f*_*SO*3, *out*−*SO*3, *in*_. Results are shown in Figure S4 in the Supplementary Material.

We thus derived three equations that will help explore the biochemistry of sulfite uptake and sulfite reduction. Equations (17) and (19) capture the role of thermodynamics in establishing degree of reaction reversibility for each of these steps. Equation (15), on the other hand, depicts their relationship for given net sulfur isotope effects. Each of these mathematical expressions heavily depends on specific biochemical parameters, which we set out to explore. They include the ratio of reduced to oxidized electron carrier complex and Gibbs free energy during sulfite reduction, and half-saturation constant and maximum reaction rate during sulfite uptake. A sensitivity analysis of the effect of each of these yields a large range of solutions over a large variable space. We then next aim at determining the subspace of tested variables that produce solutions for *f*_*SO*3, *out*−*SO*3, *in*_ and *f*_*SO*3, *in*−*H*2*S*_ that satisfy all three expressions, within the context of observed magnitude of sulfur isotope fractionation produced by the ΔQmo mutant during the chemostat experiments. For this, we aim at minimizing the residual between *f*_*SO*3, *out*−*SO*3, *in*_ calculated using 15 and those determined with Equation (19). These are independent, and the latter expression (Equation 15) is informed by *f*_*SO*3, *in*−*H*2*S*_, itself determined by Equation (17), as well as net and step-specific isotope effects.

**Figure 4:**
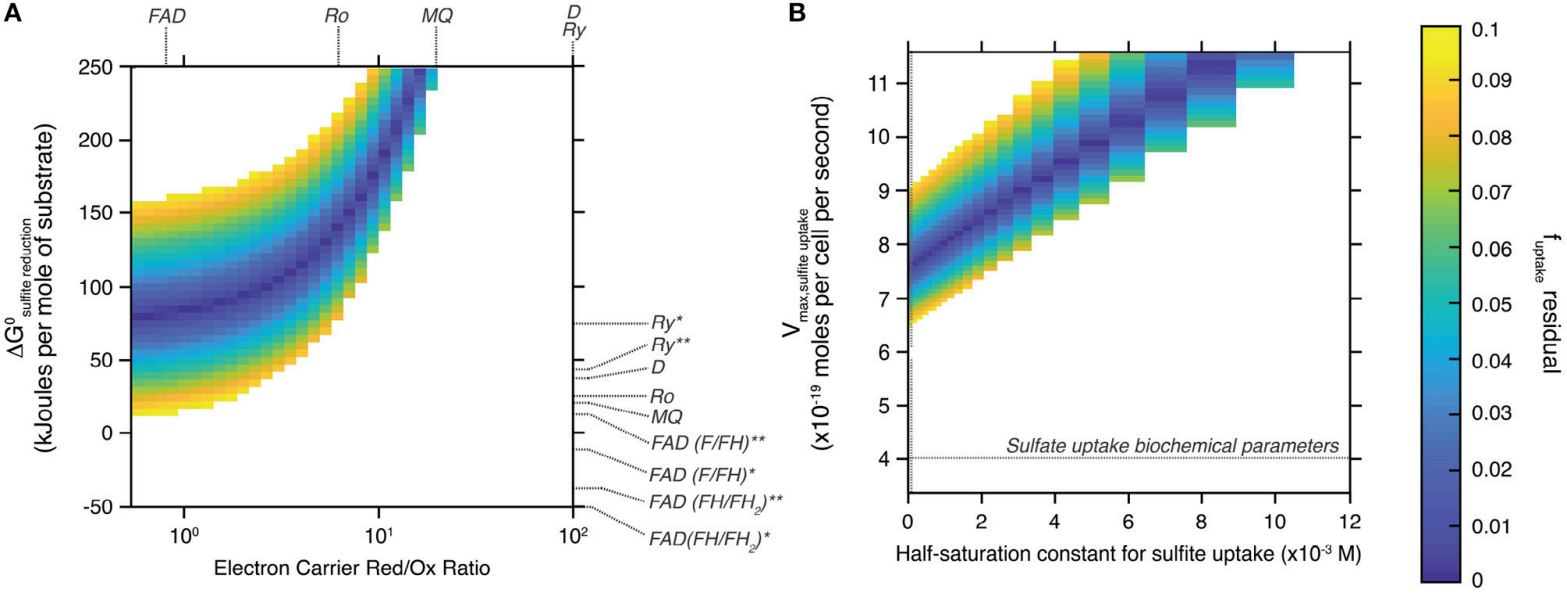
Space of solutions for the biochemical parameters that best explain the sulfur isotopic signature produced by the ΔQmo mutant. Solutions shown are those for which the difference between the two estimates of *f*_*SO*3, *out*−*SO*3, *in*_ is less than 0.1 (color-bar on the right-most side of the figure). **(A)** Shows said solutions focusing on the standard free Gibbs energy (ΔG^*o*^) of the sulfite reduction step, and the relative abundance of reduced and oxidized electron carrier compounds (Electron Carrier Red/Ox Ratio). Also shown are these same parameters for a number of common electron carriers [FAD, flavodoxin; MQ, menaquinone; Ro, rubredoxin; Ry, rubrerythrin; D, Wing and Halevy model (Wing and Halevy, 2014) default values]. The effect of the nature of the flux of electrons transferred by these electron carriers is also explored by adjusting the corresponding standard free Gibbs energy: *the redox compound is the only electron donor; **the redox compound transfers the first two electrons then the remaining four come from menaquinol oxidation. **(B)** Shows said solutions this time focusing on the biochemical parameters for sulfite uptake, that is the maximal metabolic rate of the step (V_*max, sulfiteuptake*_) and the half-saturation constant of the reaction step.

Figure 4 shows the field of solutions for which the difference between the two estimates of *f*_*SO*3, *out*−*SO*3, *in*_ is less than 0.1. Results are shown for the tested range of standard Gibbs free energy and electron carrier red/ox ratio during sulfite reduction (Figure 4A), and of maximal metabolic rate and half-saturation constant during sulfite uptake (Figure 4B). This data representation allows identifying specific values for the tested variables to simultaneously satisfy both the isotopic signature produced by the ΔQmo mutant and its specific metabolic reaction network. Also plotted in Figure 4A are the standard Gibbs free energy and electron carrier red/ox ratio combinations that correspond to different electron carriers with known biochemical characteristics (Wenk et al., 2017).

#### 4.3.3. Identifying Redox Pairs

The field of solutions produced by our analysis helps understand the biochemistry of sulfite reduction in the ΔQmo mutant metabolic pathway, and compare it to that of wild-type microbial sulfate reduction. To maintain consistency between the isotopic signature produced by this mutant, and its characteristic reaction network, sulfite reduction appears to require largely positive ΔG° values, and co-varying ratios of reduced to oxidized electron carrier abundances (Figure 4A). This trend is logical if one examines the structure of Equation (17). To maintain a given value of *f*_*SO*3, *in*−*H*2*S*_, set by the sulfur isotopic signature produced during the pure culture experiments, and render the reaction favorable, the ratio of reduced to oxidized electron carrier compounds decreases as ΔG° drops. The strong dependence between these two biochemical parameters allows narrowing down the likely electron carrier compounds responsible for the transfer of electrons during sulfite reduction. Of all candidates tested, flavodoxin was the only one with biochemical parameters that overlapped with the field of solutions. Specifically, only the case-scenario in which flavodoxin transfers the first two electrons, with the remaining four coming from menaquinol oxidation, satisfied the field of solutions (Wenk et al., 2017). The overlap is however not perfect (it only matches for *f*_*uptake, residual*_ values of 0.1), and should be taken with a grain of salt as information on the relative abundance of reduced and oxidized electron carriers is uncommon. Nonetheless, this result is consistent with work by Wenk et al. (2017), who performed an extensive sensitivity analysis on the effect of different electron carriers on net sulfur isotope fractionations for a fully constituted sulfate reduction metabolism under low energy/low sulfate reduction rates conditions. In short, it was found that under low energy-low rates of sulfate reduction conditions (that consequently induced large sulfur isotope effects), microbial sulfate reduction can proceed only with electron carriers with modestly negative reduction potential, such as rubredoxin (E′^*o*^ = −57 mV) and rubrerythrin (E′^*o*^ = 23 mV). When conditions become more favorable, sulfate reduction rates increase leading to dampened sulfur isotope effects, and a shift in metabolic strategy to employing electron carriers with strongly negative reduction potentials, including flavodoxins (E′^*o*^ = −115 mV).

#### 4.3.4. Refining Sulfite Uptake Rates

The biochemistry of sulfite uptake exhibited a strong interdependence between maximal metabolic rate and half saturation constant for sulfite uptake (Figure 4B). Overall, the field of solutions required maximal metabolic rates that exceed that of sulfate reduction by a factor of two (horizontal dotted line in Figure 4B). The corresponding half saturation constant for sulfite uptake also required large values, however the range of solutions did overlap with K_*m*_ values characteristic of sulfate uptake (vertical dotted line in Figure 4A). This difference might stem from the complex chemistry of the sulfite system (Eldridge et al., 2016). Indeed, the relative abundance of different sulfite species is a function of pH, temperature, and ionic strength. At circumneutral pH, as is the case for our system to ensure best growth of the ΔQmo mutant, the pool of aqueous sulfite available for uptake and ensuing reduction is in fact composed of a number of sulfite species, including bisulfite, and sulfite (Beyad et al., 2013). Further, bisulfite exists in two isomeric forms: a tetrahedral and a pyramidal form (Golding, 1960; Connick et al., 1982; Horner and Connick, 1986; Littlejohn et al., 1990; Risberg et al., 2009). The implication is that, at this pH boundary, the cell is likely taking up more than one species of sulfite, each with its own overall structure, size, and charge distribution. Considering the uptake of sulfate takes place via symporters that effectively dissipate a membrane proton gradient with each sulfate molecule taken up, it is not surprising that changes in the nature of the molecule being brought into the intracellular environment leads to changes in the biochemistry of this step. This is of course under the assumption that sulfite and sulfate uptake proceed via the same transporters, for which there is no definite evidence.

## 5. Conclusions

Our capacity to infer paleo-environmental conditions using the sulfur isotopic signature produced by microbial sulfate reduction heavily relies on an accurate and quantitative understanding of the metabolic underpinnings of this pathway (Harrison and Thode, 1958; Canfield, 1998; Sim et al., 2011a; Leavitt et al., 2013). The current study focuses on the main and terminal reductive step within MSR - sulfite reduction (Santos et al., 2015). We quantified the sulfur isotopic signature produced by a deletion mutant strain of the model sulfate reducer *Desulfovibrio vulgaris* (strain Hildenborough) in open system conditions at 10% of maximum growth rate, chosen to assess the upper limit of this cellular-scale isotope effect. This mutant is missing its QmoABC complex, and its metabolic network exclusively comprises sulfite reduction to sulfide. We then use an established quantitative model that incorporates net sulfur isotope effects, thermodynamics, and microbial kinetics, to explore the intrinsic biochemistry of both sulfite uptake and sulfite reduction.

The open system experiments effectively captured net sulfite reduction to sulfide. The sulfur isotopic effect produced by this isolated pathway (^34^ε = −15.9 ± 0.4 ‰) exhibited strong similarities to the case when net fractionations are set equal to the kinetic isotopic effect associated with sulfite reduction, reflecting the similarity between the experimental results of the ΔQmo mutant experiment and that with pure DsrAB (Leavitt et al., 2015). We implement these isotopic signatures and the architecture of this modified reaction network into a previously established model linking isotope fractionation and microbial biochemistry to gain a refined understanding of the mechanism of sulfite uptake and its subsequent reduction. Specifically, we find that the most likely electron carrier involved during sulfite reduction is flavodoxin, and more precisely, that this compound only performs part of the transfer of electrons, with the remainder coming from menaquinol oxidation. This is in line with previous work aiming at understanding the specific metabolic strategies employed by sulfate reducing bacteria under different energy regimes that argues for employing electron carriers with strongly negative reduction potentials, including flavodoxins (E′^*o*^ = −115 mV) under high-energy, high-sulfate reduction rate conditions. Our analysis showed the maximal metabolic rate of sulfite uptake doubles that of sulfate uptake. We argue this might be due to the complex chemistry of the sulfite system, which has direct implications for the kinetics of its uptake into the intracellular environment, assuming this transport occurs via the same symporters as for sulfate uptake. The current study established quantitative links between whole cell biochemistry, enzyme kinetics, and sulfur isotope effects for sulfite reduction. These results further our understanding of the biochemistry of the sulfite uptake and reduction steps, however the true test of the predictions made here will be a series of carefully calibrated experiments, guided by the outcome of our combined experimental and modeling approach. This is necessary to further inform the interpretation of geological and paleo-environmental records, and our understanding of secular changes to Earth's surface redox conditions.

## Author Contributions

EB ran the chemostat experiments, the substrate abundance and substrate-specific isotopic analysis, adapted the metabolic model to this specific system. GZ and JW produced the deletion mutant central to this study. BW and IH developed the metabolic model used in the study. All authors contributed to the writing of the paper.

### Conflict of Interest Statement

The authors declare that the research was conducted in the absence of any commercial or financial relationships that could be construed as a potential conflict of interest.
