# Supplementary material for: Deconstructing the Dissimilatory Sulfate Reduction Pathway: Isotope Fractionation of a Mutant Unable of Growth on Sulfate
Source: Front Microbiol. 2018 Dec 14;9:3110. doi: 10.3389/fmicb.2018.03110 (PMC6302107; doi:10.3389/fmicb.2018.03110)
Supplement: Supplementary file 2 [file Data_Sheet_1.pdf]

Supplementary Material

Microbial experimental procedures

Growth Media JW9021 was grown anaerobically at 24°C in a freshwater medium of the following composition: 8 mM $MgCl_2$, 20 mM NH_4Cl, 0.6 mM $CaCl_2$, 2 mM K_2HPO_4-NaH_2PO_4, 6 mL/L of a trace metals solution, 30 mM Tris-HCl (pH: 7.4), and 1 mL/L of a 10X Thauers vitamins solution (Grant et al, 2010). Sterility prior to inoculation was achieved via autoclaving. Anaerobic conditions were achieved by maintaining the chemostat under positive pressures of N_2:CO_2 (90:10, 5 psi). The final lactate and sulfite concentrations in the medium delivered to the reactor were 10 and 20 mM, respectively.

Chemostat materials All surfaces in the chemostat device consisted of glass, polyether ketone (PEEK) or plytetrafluoroethylene to avoid reoxidation of biogenic sulfide (either in aqueous or gaseous form). The reactor vessel was a six-port, 3-L working volume vessel (1964-06660, Bellco Glass), its pH was kept constant (7.2 ± 0.1) by means of a pH probe-activated titration pump (DLX pH-RX/MBB metering pump; Etatron), dosing 1 M HCl, previously degassed with N_2 and autoclave-sterilized. Input and output fluxes were set at the same rate using a single pump (Ismated four0channel Reglo analog peristaltic pump with Tygon HC F-4040-A tubing).

Analytical methods

Chemical methods Lactate and acetate concentrations were determined via anion chromatography with conductivity detection using an eluent gradient method (ICS-2000, AS11 column, Dionex). Sulfite, sulfide and thiosulfate concentrations were determined using the fuschin, methylene blue and the cyanolysis assays, respectively. All colorimetric readings were made using a UV-Vis Spectramax Plus 384 plate reader. Standards were run alongside samples in each assay every time for purposes of calibration. Cell densities were monitored at optical densities of 600 nm using a UV-Vis Spectramax Plus 384 plate reader (Molecular Devices).

Sulfur phase separation Each sulfur pool of interest was separated and purified prior to isotopic analysis, which is a sequential extraction and purification of each sulfur pool. Sulfides and sulfites found in solution were precipitated and separated as zinc sulfide and barium sulfite via filtration. Thiosulfate left in the supernatant was decomposed to silver sulfide (bearing the sulfane sulfur) and barium sulfate (bearing the sulfonate sulfur moiety) upon addition of excess silver nitrate. Sulfur was extracted from each phase by means of Acid Volatile Sulfur (AVS) (for sulfides) and Thode extractions (for sulfates).

Figures

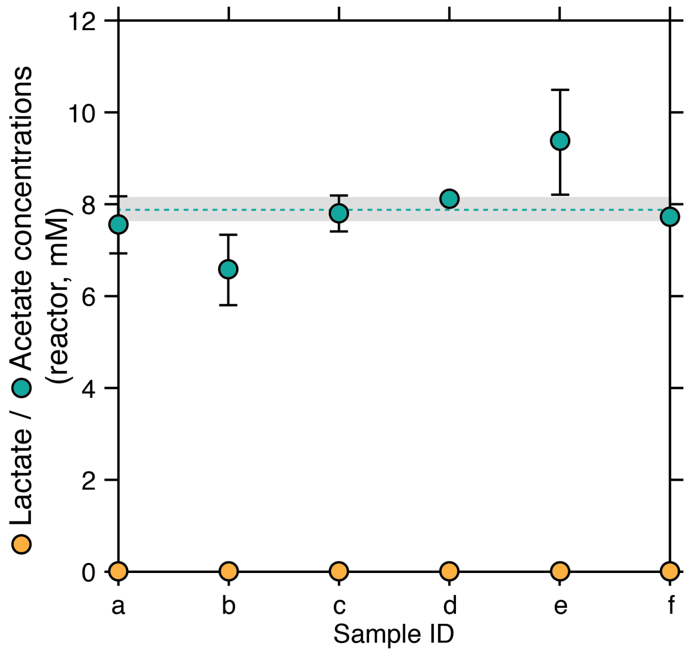

Figure 1: Concentrations of lactate (yellow circles) and acetate (blue circles) in the reactor for each sampling point, determined via ion chromatography, in units of mM. Vertical lines correspond to the 1 σ for each sampling point. Average values for acetate levels in the reactor across sampling events is shown as a dotted line, and the corresponding error (1 σ, after propagation of sample-specific error) is shown as the gray area around the dotted line.

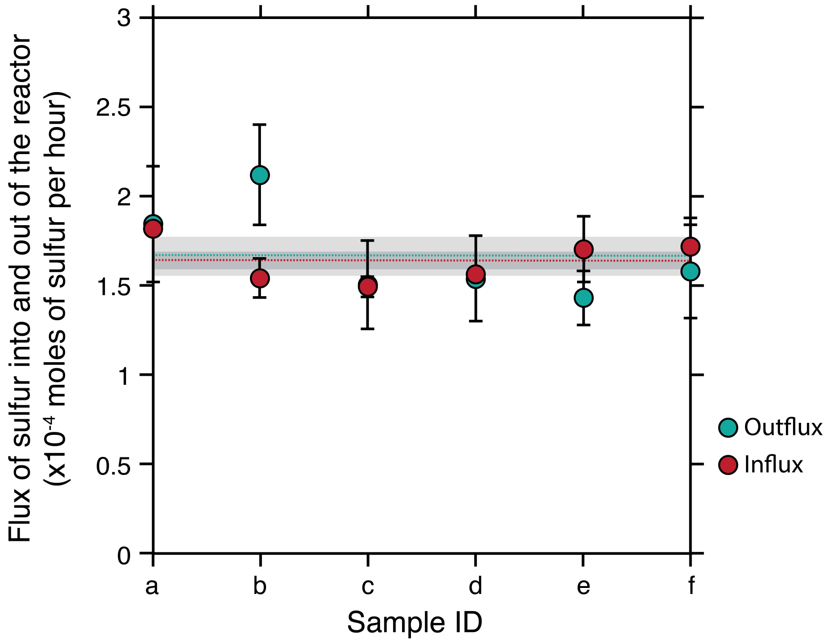

Figure 2: Total sulfur entering (red circles) and leaving (blue circles) the reactor (in units of moles per hour) for each sample taken over the reported time interval, and the corresponding averages (dotted lines, red for influx, blue for outflux).

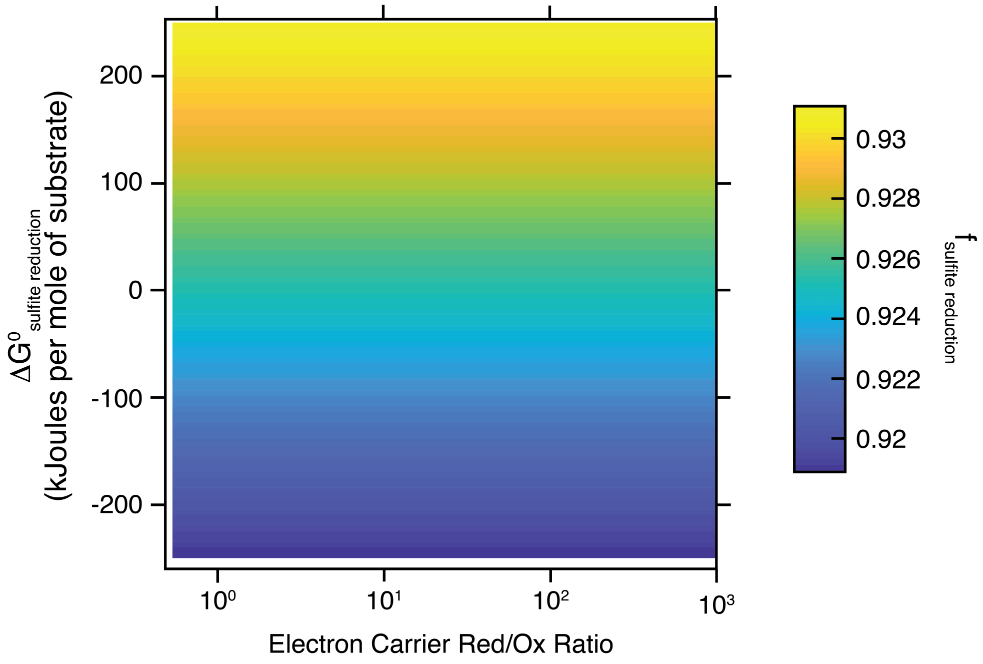

Figure 3: Sensitivity analysis of the effect of the standard free Gibbs energy (ΔG^o) of the sulfite reduction step, and the relative abundance of reduced and oxidized electron carrier compounds (Electron Carrier Red/Ox Ratio) on the degree of reversibility of sulfite reduction ($f_{SO3,in-H2S}$), calculated according to equation 17.

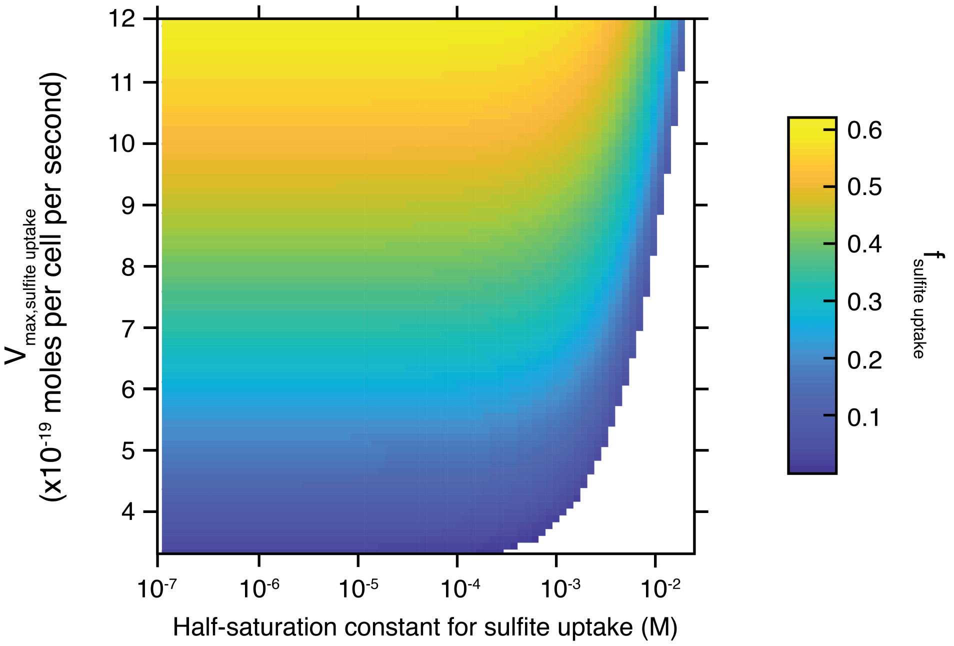

Figure 4: Sensitivity analysis of the effect of the biochemical parameters for sulfite uptake, that is the maximal metabolic rate of the step ($V_{max,sulfiteuptake}$) and the half-saturation constant of the reaction step on the degree of reversibility of sulfite uptake ($f_{SO3,out-SO3,in}$), calculated according to equation 19.

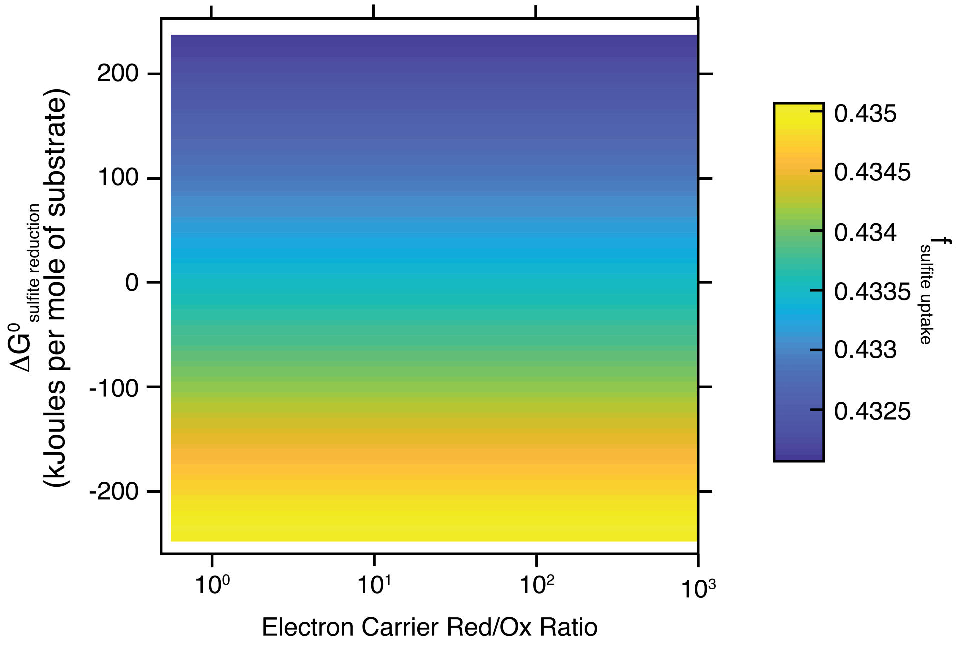

Figure 5: Corresponding values for the degree of reversibility of sulfite uptake ($f_{SO3,out-SO3,in}$) using $f_{SO3,in-H2S}$ and equation 15.
